# Proteomics Based Identification of Cell Migration Related Proteins in HBV Expressing HepG2 Cells

**DOI:** 10.1371/journal.pone.0095621

**Published:** 2014-04-24

**Authors:** Huixing Feng, Xi Li, Vincent Chan, Wei Ning Chen

**Affiliations:** School of Chemical and Biomedical Engineering, Nanyang Technological University, Singapore, Singapore; Rutgers, The State University of New Jersey, United States of America

## Abstract

Proteomics study was performed to investigate the specific protein expression profiles of HepG2 cells transfected with mutant HBV compared with wildtype HBV genome, aiming to identify the specific functions of SH3 binding domain (proline rich region) located in HBx. In addition to the cell movement and kinetics changes due to the expression of HBV genome we have observed previously, here we further targeted to explore the specific changes of cellular proteins and potential intracellular protein interactions, which might provide more information of the potential cellular mechanism of the differentiated cell movements. Specific changes of a number of proteins were shown in global protein profiling in HepG2 cells expressing wildtype HBV, including cell migration related proteins, and interestingly the changes were found recovered by SH3 binding domain mutated HBV. The distinctive expressions of proteins were validated by Western blot analysis.

## Introduction

Hepatocellular carcinoma (HCC) is the third fatal reason of cancer related death in the world. The five years survival rate for advanced HCC is lower than 5% [1]. Considering most HCCs are detected at an advanced stage, the incidence and the mortality rates are similar. Hepatitis B virus, together with hepatitis C virus infection is the two primary causes for liver cancer and other liver diseases. HBV infection, due to the high prevalence compared with HCV, outweighs HCV as the most clinico-epidemiologically important risk factor of HCC [2].

Hepatitis B virus genome is circular and partially double stranded DNA. It is composed of around 3200 base pairs with slight differences among the eight genotypes [3]. The four overlapped open reading frames encode polymerase, the core and e-antigen, HBx and the pre-S/S gene which encodes the three surface antigens [3]. HBx is a small protein composed of 154 amino acids. Among all HBV genes, HBx is the most critical carcinogenic component [4]. No information of 3-dimensional structure is available for HBx up to now, which has put much difficulty in the understanding of its function. However, HBx has been well accepted as a multifunctional gene and plays an important role in the development of HBV related liver diseases. It has been reported as transcriptional regulator and it is believed to participate in many intracellular signal pathways, such as signal transduction, apoptosis, cell cycle progression and protein degradation pathways through interaction with host genes. Although numerous work has been done in many groups in the past years, the knowledge of HBx is not comprehensive, especially the specific molecular mechanism through which HBx interacts with host cell factors.

Together with other characteristic domains, SH3 domain and binding domain intermediated protein-protein interaction is broadly existed in the cellular signal transduction. In our previous work, we identified a proline rich domain in HBx, which was also recognized as SH3 binding domain. The SH3 binding array assay showed this region was able to bind with a number of proteins with SH3 domain [5], [6]. In our HBV induced HepG2 cell deadhesion and migration assay, the proline amino acids were selectively mutated to alanine targeting to disfunction the SH3 binding domain. The cell movements were tracked and cell deadhesion and migration kinetics was focused. The HepG2 cells with mutant HBV expression showed different migration scenario compared with the cells expressing wildtype HBV. Particularly, the increased cell movement was shown recovered by the expression of proline mutated HBV genome [7]. The results might imply that the proline rich region played an important role in the virus and host cell interaction that regulating the cell movements. To find out the possible responding factors in the cells, in this work, by applying the same mutant genome, we further focused on the overall protein expression profile aiming to explore the possible mechanism of cell-cell interaction and regulation intermediated by SH3 binding domain located in HBx.

In the past several years, the “-omics” approaches, especially proteomics have been employed for the systematic research. These methods are particularly useful when applied for the screening of biomarkers for diseases progression. Combined with clinical diseases outcomes, those biomarkers could provide useful information for early diagnosis of fatal diseases such as cancers. Moreover, the proteomics based systematic research could also provide extensive information in identifying potential protein-protein interactions, signal pathways and in turn the possible reason and mechanism of diseases development and progression. Continuing our previous work, to further understand the mechanism of altered movements of hepG2 cells expressing HBV genome, here we aim to explore and compare the global protein expression profiles of cells expressing wildtype HBV and mutant HBV, the iTRAQ-labeling (Isobaric tag for relative and absolute quantitation) facilitated quantitative proteomic analysis was performed.

## Materials and Methods

### 1. Plasmids and Cell Lines

The plasmids with wild type replicative HBV genome (pcDNA3.1-wtHBV) and mutant replicative HBV genome (pcDNA3.1-mHBV) were constructed previously as described [7]. To mimic the in vivo environment, the replicative HBV genome was constructed into mammalian expression vector pcDNA3.1, instead of the circular format, the genome was constructed in the form of linear DNA. A terminal redundancy of around 0.1 genome length was applied for the intact protein transcription of the overlapped organized genome [8].

The site mutations were introduced with commercial site directed mutagenesis kit (QuikChange XL, QIAGEN, USA) and specifically designed primers with proline to analine mutations in SH3 binding domain existed in HBx [7]. The empty expression vector pcDNA3.1 was used as negative control.

HepG2 cells were obtained from ATCC. The cells were cultured with Gibco Dulbecco's minimal essential medium (MEM) (Invitrogen Inc., USA), complemented with 10% fetal bovine serum and 1% anti-mycotic (All come from Invitrogen Inc., USA) under 37°C and 5% CO_2_. The cells were passaged by trypsinization with 0.1 M Trypsin-EDTA/PBS (pH 7.2) at 37°C (Invitrogen Inc., USA) every 3–4 days.

### 2. Cell Transfection

The transfection was performed by applying Nucleofection solution V (Lonza, Switzerland). Approximately, 2.5×10^6^ HepG2 cells were seeded in 10 cm petri dishes 24 hours prior to transfection, and 2–5 µg (according to the number of cells) plasmids were transfected following the manufacturer's instruction. Shortly, trypsinized HepG2 cell pallets were resuspended carefully in 100 µl transfection solution V provided by the kit and the plasmids were added in. Then the mixture was moved into kit provided sterile cuvettes. Necleofection was performed with inner set program specific for HepG2 cells. Then 500 µl medium pre-warmed to 37°C was immediately added into the cuvette and the mixed solution was transferred to 10 cm petri dish with prewarmed medium. Four hours post transfection, the medium with un-adhered cells was removed with the fill of fresh medium. The transfected HepG2 cells were cultured for 2 days before collection.

### 3. Protein Extraction and Quantitation

The harvested cells were resuspended in 300 ul lysis buffer (8 M urea, 4% (w/v) CHAPS, and 0.05% SDS (w/v)), kept on on ice for 30 min with regular vortex followed by centrifugation at 15,000 g at 4°C for 1 hour to remove the insoluble cell debris. The supernatant was transferred out, and the concentration of protein in each sample was measured with the Bradford Protein Assay (Bio-Rad, US). The cell lysates were kept in −80°C for further experiments.

### 4. Protein Digestion and iTRAQ Labeling

For each sample, peptides digested from 100 µg proteins were used for iTRAQ labeling. The proteins were precipitated with 4 volumes of cold acetone at −20°C for 2 hours and centrifugated for 10 minutes at 15,000 g. Then the protein precipitates were dissolved in the dissolution buffer, followed by reduction and alkylation with TCEP and MMTS provided by the iTRAQ Reagent Multiplex Kit (Applied Biosystems, CA). The proteins were then digested with 20 µL of 0.25 µg/µL sequence grade modified trypsin (Promega, US) solution at 37°C for 12–16 hours. Peptides digested from the cells transfected with empty vector pcDNA3.1, pcDNA3.1-wtHBV, pcDNA3.1-mHBV were individually labeled with the iTRAQ reagents with isobaric tag of 114, 115 and 116 respectively. The labeled three samples were mixed, dried and resuspended in 300 µl solution (95/5 of ddH_2_O/Acetonitrile, 0.1% Formic acid) to get 1 µg/µl final concentration roughly. The iTRAQ labeled sample was stored in −80°C.

### 5. On-line 2D LC-MS/MS Analysis

The analysis was performed with online two-dimensional system, which was composed of 1200 Series liquid chromatography, Chip-Cube interface system, and 6530 Quadruple - Time of Flights mass spectrometer (all Agilent Technologies, CA). Briefly, the samples were separated and analyzed by strong cation exchange column (SCX column) and nano-LC-chip. The SCX column was manually equipped in the system. The chip (Agilent Technologies, CA) was integrated with an enrichment column (large capacity, 160-nl) and a 150 mm×75 µm C18 reverse phase column (Zorbax 300SB 5 µm).

Firstly, 2∼5 µg iTRAQ labeled peptides were loaded onto and separated by PolySulfoethyl A strong cation exchange nano-column (0.32×50 mm, 5 µm). The peptides were sequentially eluted by injection of gradient ammonium format solution with concentrations of 20, 40, 60, 80, 100, 200, 500, 1000 mM. During every injection, the peptides eluted from strong cation exchange column were brought into the nano-LC-chip. The peptides were retained in the nano enrichment column first, followed by the analysis with C18 reverse phase column, which was also integrated in the nano-LC-Chip. The peptides were loaded onto the enrichment column with 97% solvent A (ddH_2_O/0.1% formic acid) at a flow rate of 4 µl/min. Then the HPLC-chip automatically switched to analysis mode from enrichment mode, and the retained peptides were eluted with gradient mixture of buffer A (ddH_2_O/0.1% Formic Acid) and buffer B (Acetonitrile with 0.1% Formic Acid) at a glow rate of 0.3 µl/min. The nanoflow gradient was from 5% to 50% buffer B over 45 min, then the concentration of buffer B was increased to 90% B by 5 min. The flow was kept for 5 min, and then brought to initial state in 10 min for reconditioning the column.

The effluent from analysis column of the chip was directly analyzed by Q-TOF mass spectrometer that was interfaced in tandem through a nano-LC-Chip Cube nanospray source. Agilent MassHunter Workstation version B.02.01 was used for the equipment controlling and MS data acquisition. The chip-cube was operated at a capillary voltage of 1800–2300 V (depending on the condition of the chip) for stable and optimistic spay. For peptide analysis, positive ionization mode was used for the data acquisition. The MS was acquired from m/z 450 to 1500, and up to four most abundant ions (with charge states 2+, 3+, and >3+) were selected as precursors for further MS/MS analysis in each cycle. Reference ions of 121.005 and 922.0098 were used for reference mass correction.

### 6. LC-MS/MS Data Analysis

MS/MS generated spectra were deconvoluted and analyzed using Spectrum Mill, version Rev A03.03.084 (Agilent Technologist, CA). The peptides were identified against UniProtKB/Swiss-Prot protein database (Geneva, Switzerland) for species of *Homo Sapiens*. The searches were run with the following settings: methylmethanethiosulfate-labeled cysteine and iTRAQ modification at the amino group were set as fixed modification; methionine oxidation was set as variable modification; mass tolerance for precursor ions: 20 ppm; mass tolerance for fragmented product ions: 50 ppm; maximum ambiguous precursor charge: 5; two missed cleavages were allowed.

Auto-validation was used for the searching and those criteria ensure high confidence for the identified proteins that always represent valid data. The same criteria were applied for the analysis of each independent sample. The quantitation of iTRAQ labeled proteins was based on the MS/MS spectrals and the results were presented as the ratios of the areas under each isobaric reagent tags. The relative amount of a single peptide in the sample was obtained by comparing the peak areas at 115.1 and 116.1 with that at 114.1 m/z and the ratios could be calculated. The ratios were corrected with overlapped isotopic contributions and applied to calculate the relative abundances of a particular peptide. For proteins with more than one qualified peptide matches, the peak area ratios of the peptides originating from the same protein were considered to calculate the average peak area ratios.

### 7. Bioinformatics Analysis

The molecular functions of proteins were batch searched and cataloged by PANTHER classification system (http://www.pantherdb.org/) against UniProtKB (*Homo Sapiens*) database (released at 2013_01).

### 8. Western Blot ananlysis

Western blot was performed as routine procedure. The transfected cells were harvested with cell lysis buffer followed with gentle sonication. Normally, the proteins were separated with 10%–15% SDS-PAGE. The proteins were transferred onto either polyvinylidene difluoride (PVDF) membrane or nitrocellulose (NC) membrane (GE, USA) with semi-dry transfer apparatus (Bio-rad, US). The proteins were detected with respective antibodies. The dilutions of primary antibodies were optimized according to the original concentrations and sensitivity, ranging from 1∶1000 to 1∶10,000. In this work, Mouse anti-β-actin (AC-74, Sigma), mouse anti-IQGAP1 (sc-374307, Santa Cruz), rabbit anti-PDIA3 (HPA003230, Sigma) and mouse anti-Annexin A2 (C-10: sc-28385, Santa Cruz) were used as primary antibodies. HRP-conjugated anti-mouse IgG (#31430, Pierce) and ImmunoPure Goat Anti-Rabbit IgG (H1L), peroxidaseconjugated antibody (31460, Pierce) were used as secondary antibodies. SuperSignal West Pico Chemiluminescent reagent (Thermo scientific, US) was uesd for enhanced Chemiluminescence (ECL), and detection was performed by using CL-XPosure Film (GE Healthcare, UK).

### 9. Statistical Analysis

Five independent biological repeats were carried out. Only proteins appeared in at least three repeats with consistent changes were accounted and presented here.

## Results

### 1. Wild Type and Mutant HBV Expression in HepG2 Cells

**Figure 1:**
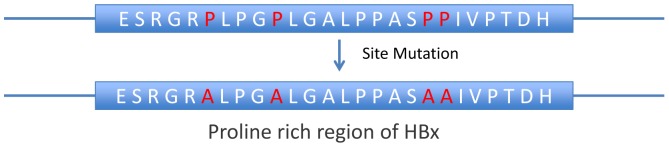
Diagram of proline rich region site mutations. Four prolines were mutated into analines to break the canonical PXXPPXXP SH3 binding domain located in HBx of HBV genome.

The mutated full length HBV genome on mammalian expression vector pcDNA3.1 was generated previously as described [7]. Four prolines were site mutated into alanines to disfunction the proline rich region as shown in Figure 1. The successful transfection and expression were validated by co-transfection with GFP (Green Fluorescent Protein) and RT-PCR (Reverse Transcriptional Polymerase Chain Reaction) [7].

### 2. iTRAQ Labeling Based 2D Proteomics Analysis and Differentially Identified Proteins

**Figure 2:**
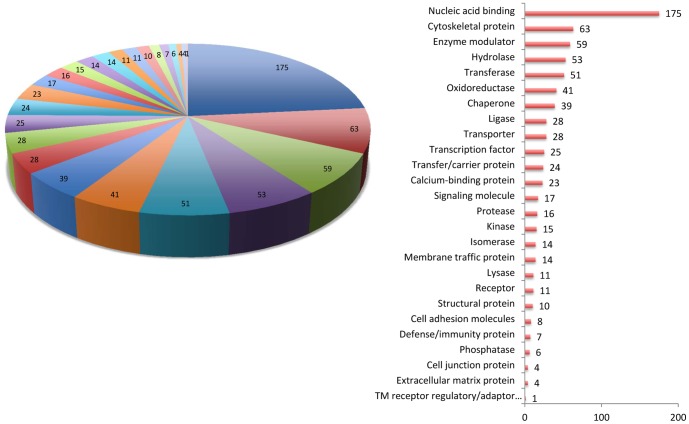
Classification of quantified proteins by iTRAQ labeled profiling. Gene IDs of 689 proteins were batch searched using PANTHER classification system against UniProtKB (*Homo. sapiens*). The genes were mapped to a total number of 747 hits in 26 categories.

Approximately, 700 unique proteins were identified in 5 independent biological repeats with a minimum score of 11 and SPI% of 70 as searching and validation criteria. According to the software provider's instruction, such stringent criteria set provides high confidence for the results. Only the proteins appeared in at least three independent repeats with consistent trends of changes for both of transfected cell lines when compared with control, which was wHBV/ctrl, or mHBV/ctrl, were considered in the following analysis and discussion. For all the identified proteins, a large ratio of proteins was nucleic acid binding proteins, and around 10% of the proteins are cytoskeletal proteins (Figure 2). Other groups, cell adhesion molecules, structural proteins, cell junction proteins and extracellular matrix proteins that might be highly related with cytoskeleton or cytoskeletal reorganization were also identified. A great number of cellular enzymes (hydrolase, transferase, oxidoreductase, ligase, protease and so on) and enzyme modulator were found. Twenty-three of the proteins belonged to the family of calcium binding protein. Among those proteins with consistent trends of changes, only the changing level was more 20% in at least three repeats of either group (wHBV/ctrl, or mHBV/ctrl) were discussed here. With this standard, 17 proteins were considered as significantly changed and listed here (Table 1). These proteins were found perturbed by the expression of wildtype HBV genome, the changes were recovered in different distances by the mutation of proline rich region in HBx.

**Table 1:** List of differentially expressed proteins in HepG2 cells expressing wHBV and mHBV compared with empty vector control.

Accession No.	Times Identified	No. of Peptides	Protein Name	Biological Process and Function	Ratios	
					wHBV/Ctrl	mHBV/Ctrl
P07437	5	14	Tubulin beta chain	Structural constituent of cytoskeleton	1.18±0.1	1.09±0.04
P23528	5	6	Cofilin-1	Structural constituent of cytoskeleton; acting binding	1.21±0.12	1.05±0.24
P46940	4	3	Ras GTPase-activating-like protein IQGAP1	Intracellular protein transport; nuclear transport; induction of apoptosis; signal transduction; protein metabolic process	1.56±0.2	1.18±0.17
Q13283	3	2	Ras GTPase-activating protein -binding protein 1 (G3BP1)	RNA binding; receptor binding; transport	1.54±0.14	1.31±0.07
P10809	5	15	60 kD heat shock protein	Protein complex assembly protein folding	1.2±0.09	1.15±0.11
P38646	5	13	Stress-70 protein	Heat shock protein, protein folding; protein complex assembly; response to stress	1.3±0.34	1.22±0.11
P30101	5	11	Protein disulfide-isomerase A3	Protein disulfide isomerase activity protein; modification process	1.34±0.3	1.26±0.26
P07355	5	8	Annexin A2	Calcium-dependent phospholipid binding; intracellular protein transport; signal transduction	1.28±0.29	1.13±0.19
Q15365	4	4	Poly(rc)-binding protein 1	Intracellular protein transport; nuclear transport; induction of apoptosis; signal transduction; protein metabolic process	1.33±0.22	1.12±0.17
Q7Z2W4	5	8	Zinc finger CCCH-type antiviral protein 1	Nucleobase, nucleoside, nucleotide and nucleic acid binding, metabolic process	1.42±0.23	1.18±0.15
P09429	3	4	HMGB1	Transcription regulation, involved in inflammation	1.28±0.28	1.03±0.05
P13639	5	13	Elongation factor 2	Translation factor activity; nucleic acid binding; translation elongation factor activity	1.33±0.14	1.16±0.09
P62987	4	5	Ubiquitin	Regulatory protein	1.15±0.15	0.95±0.12
P61769	3	2	Beta-2-microglobulin	Antigen processing and presentation; cellular defense response	1.38±0.06	1.16±0.09
P02765	3	9	Alpha-2-HS-glycoprotein	Proteolysis; mesoderm development; skeletal system development	1.23±0.13	1.03±0.14
Q15181	5	5	Inorganic pyrophosphatase	Pyrophosphatase activity	1.3±0.25	1.14±0.17
P04075	5	7	Fructose-biphosphate aldolase A	Lysase activity glycosis	1.25±0.45	1.28±0.19

### 3. Validation of Differentially Changed Proteins by Western Blot

**Figure 3:**
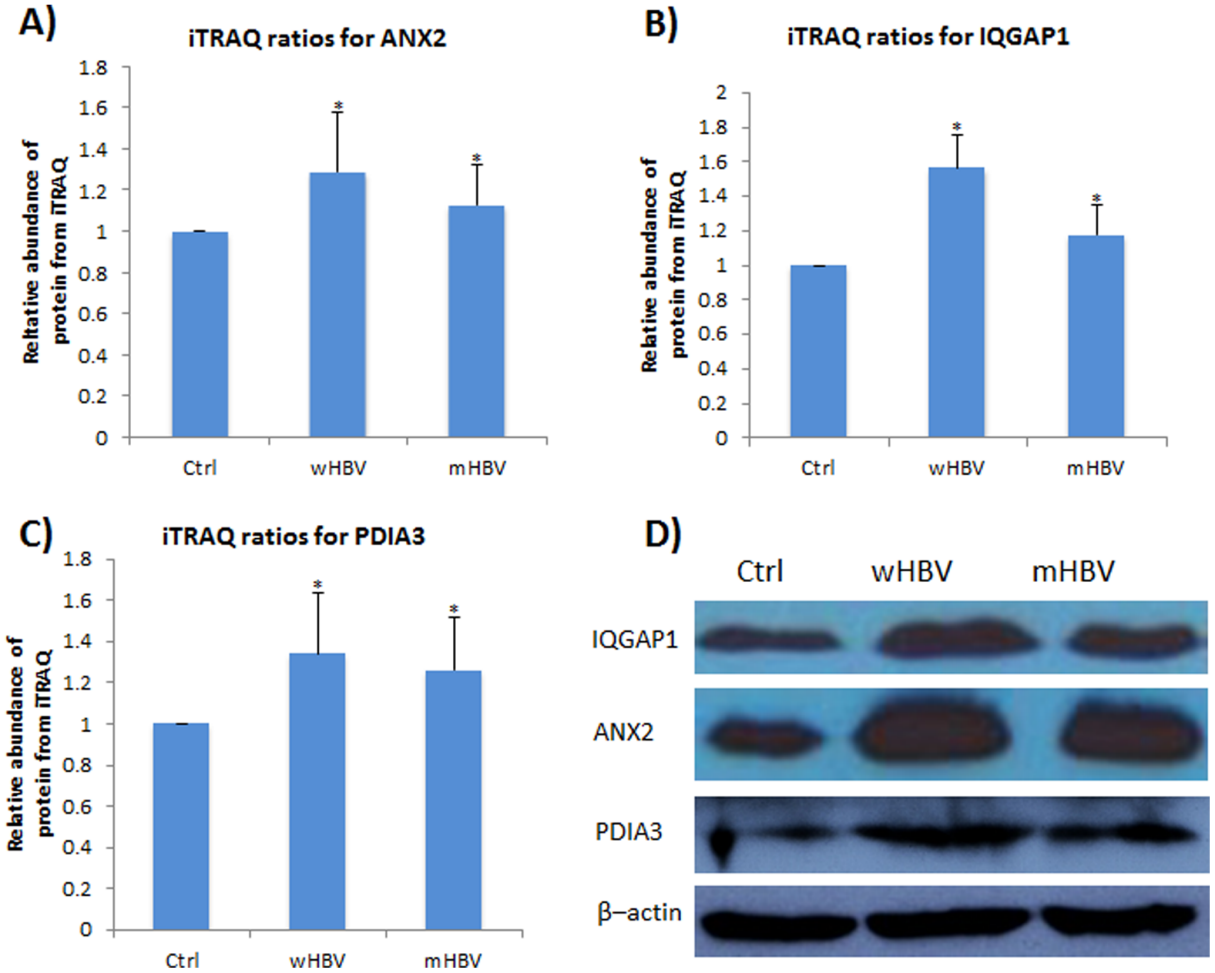
Western blot validation of selected differentially expressed proteins from the LC-MS/MS data sets. A) iTRAQ ratios for protein annexin A2; B) iTRAQ ratios for protein IQGAP1; C) iTRAQ ratios for protein disulfide isomerase A3; D) western blot validation result for annexin A2, IQGAP1and protein disulfide isomerase A3. The p-value was indicated by “asterisk”: *, p<0.05.

To validate the changes identified in proteomics profiling, Western blot was performed. Several proteins were chosen of the validation. The protein total lysate was quantitated in the same method with the samples applied for LC-MS/MS analysis. A total of around 20 ug total proteins were loaded for each sample. The quantitation was further confirmed with internal control β–actin as shown in figure 3. Three proteins, Annexin A2, IQGAP1 and protein disulfide isomerase A3 (PDIA3) were validated by Western blot.

## Discussion

The cell line based system has been popularly used in HBV-associated HCC proteomic research as a complementary strategy of clinical sample based platform due to homogenous genetic background and enhanced data reproducibility. In this work, we studied global protein expression profiling of HepG2 cell line expressing HBV genome compared with mutant HBV genome. Similarly, HBV-expressing stable cell lines and transgenic mice were applied in HBV associated HCC research by other groups [9]–[11]. The work of Wei's group has shown that a series of proteins with various functions belonging to different groups such as calcium binding proteins, retinol metabolism, and protein degradation pathways were affected by the stable expression of HBV in HepG2.2.15 cells. The work was performed on 2D-gel separation coupled with mass spectrometry protein identification, and the differentially expressed proteins were confirmed by quantitative PCR and western blot [10], [11]. In this work, online 2D nanoHPLC separation were coupled with Q-TOF MS, 17 proteins expression levels including different functions, especially cytoskeletal related proteins were found disturbed in wild type HBV containing cells.

### 1. Differentially Expressed Cytoskeletal Related Proteins

The majority of proteins list in table 1 showed upregulation in the cells expressing wildtype HBV genome, and the increased expression showed strong or moderate recovery in the cells expressing proline rich region mutated HBV genome. Among the differentially expressed proteins, some of them raised our interests considering their intracellular molecular functions.

Ras GTPase-activating-like protein 1 (IQGAP1) was found significantly upregulated (1.56±0.2) in wildtype HBV (wHBV) genome expressing cell line, and the increasing ratio was decreased in mutated HBV (mHBV) expressing cell line, although it was still higher than the control cell.

IQGAP1 is a 190 kD protein composed of 5 domains, calponin homology domain (CHD), WW domain, IQ domain, RasGAP_c carboxy terminal sequence and RasGAP-related domain (GRD) [12]. The calponin domain mediates actin-binding [13] and binds calponin. The IQ domain binds calmodulin, which is a calcium sensor protein that is involved in the interaction and regulation with a number of proteins. The GRD domain is similar with the functional subunit of GAPs (Ras GTPase-activating proteins). However, the function is different, it binds Rac1 and cdc42 and stabilizes the GTP-bound active state of Rho GTPases [14]. The C-terminal RasGAP_c carboxyl sequence is capable of binding with E-cadherin and Beta-catenin [12]. Particularly, WW domain has two functionally conserved tryptophans and it is associated with proline rich sequences of many proteins in the protein-protein interaction, which is similar with SH3. SH3 and WW domains are the two identified significant protein scaffolds to recognize ligands with proline-rich regions [15]. With these five functional domains, IQGAP1 is able to bind Rac1 and Cdc42, β-catenin, E-cadherin, calmodulin and components of the mitogen-activated protein kinase pathway, all of which are involved in cancer development.

IQGAP1 is believed to play a critical role in cell motility and invasion [16]. The overexpression of IQGAP1 in mammalian cells boosts cell migration in a Rac1- and Cdc42-dependent manner. The knock down of endogenous IQGAP1 or the expression of negative IQGAP1 construct showed significant effect in decreasing the cell motility. Cell invasion was also shown altered by the manipulation of IQGAP1 concentrations [16]. It has been observed that IQGAP1 was over-expressed and membrane localized distinctly in various tumors, such as liver, breast, lung, gastric, ovarian, and colorectal cancers [17]. In the cell line based work, it was found up-regulated in gastric cancer cell line [18].

In normal cells IQGAP1 was found be localized at the areas with rapid actin turnover. Similarly, in invasive tissues, IQGAP1 was proven to localize at the leading edge of migrating cells [19]. IQGAP1 was shown over-expressed in human breast epithelial cancer cell line and the high level of IQGAP1 showed correlation with enhanced cell migration and invasion [19], [20]. For liver cancer, the expression of IQGAP1 was found increased in HCC and it was believed to promote cell proliferation by the activation of Akt [21].

In HBx protein of HBV, we identified a proline rich region earlier, and the characteristic of which was believed to be able to bind SH3 domain and WW domain. These two functional domains played important roles during the intracellular protein-protein interaction. In our proteomics global protein profile, IQGAP1 was found greatly up-regulated in HepG2 cells transfected with wildtype HBV genome (1.56±0.2, wHBV/Ctrl), which is more than 50% increment. And interestingly, the increment was decreased to 1.18±0.17 (mHBV/Ctrl). Although in the mutated HBV expressing cell line, IQGAP1 level was still higher when compared with control, but the increment distance was much less than in the wildtype HBV cell line. This may indicate that HBV was able to interact with IQGAP1 via proline rich region and WW domain binding. And in turn the binding of IQGAP and Rho GTPases Rac1 or Cdc42 were enhanced. The increased IQGAP1 could induce stronger actin binding and Rac1 activation, which would result in cytoskeletal change, cell deadhesion and cell migration. We had demonstrated that HBx was able to activate Rac1 in previous work [6]. However, the activation pathway was not clear.

Another Rho GTPase related protein, Ras GTPase-activating protein-binding protein 1 (G3BP1), was also found upregulated. G3BP1 is also recognized as Ras-GTPase-activating protein SH3-domain-binding protein. It was reported of the binding to the SH3 domain of Ras GTPase-activating protein in proliferating cells, however, in quiescent cells, no interaction was observed [22]. G3BP1 was found interacting with HCV non-structural proteins and RNA. Data from immunoprecipitation and pull down assay suggested that G3BP1 might be a component of HCV replication complex and function in HCV genome amplification [23]. The knockdown of G3BP1 significantly reduced virus replication in HCV replicon cells [24]. G3BP1 was also found to play an important role in RNA replication process of Sindbis virus, via association with the non-structural protein of the virus [25]. However, little information could be found about the relationship of this protein and HBV and HBV caused liver cancer, the up-regulation of this protein may due to the increased proliferation of HepG2 cells transfected with viral genome. Our finding of the elevated level of this protein may lead to new clue in illustrating the HBV replication mechanism via the SH3 binding domain.

Cofilin 1, another cytoskeletal organization protein, was also found disturbed in HepG2 cells transfected with wildtype HBV genome. Cofilin 1, also named CFL1, belongs to the cofilin family. It is a widely distributed in the cell and plays an important role in the intracellular actin modulation. By the binding and depolymerizing of filamentous F-actin, cofilin was found stimulating filament disassembly. Similar with IQGAP1, cofilin promotes high actin turnover [26]. It was reported that the consequenced phosphorylation of cofilin from the phosphorylation of LIM-kinase by Rho kinase (ROCK), which could be activated by Rho, contributed to action reorganization induced by Rho [27]. Similarly, phosphorylation of cofilin could also be induced by PAK, a downstream effector of Rac1 [28].

Annexin A2 was found upregulated and the increment was recovered by the mutated HBV. Annexin A2 (ANX2) is a member of Annexin family, which is composed of members of calcium dependent proteins that widely existed in nearly all eukaryotes. They show critical functions in wide range of cellular activities, diverse signal transduction pathways and the regulation of cellular growth. Annexin A2 is a pleiotropic protein and it is associated with a vast number of cellular processes such as apoptosis, proliferation, fibrinolysis, cell matrix interactions, cell migration, invasion and adhesion [29]. Altered expression of ANX2 has been identified in various cancers, such as pancreatic cancer, breast cancer, brain cancer, and liver cancer [30]. In the work of brain cancer research, it was believed that the increased plasmin activity on the tumor cell surface could mediate the degradation of extracellular matrix (ECM) and promotes neo-angiogenesis, which in turn facilitates tumor growth [31]. It was also found elevated in early stage of HCC, in accordance with the finding in HCC–bearing nice [32]. In our HBx expressed HepG2 cell line, we found that ANX2 was up-regulated [33]. Similarly, in this work, ANX2 was consistently upregulated and the enhanced expression was slightly recovered by the mutation of HBx proline rich region. Overall speaking, HBx and/or HBV expression similarly increased the level of ANX2 and the effect of HBV SH3 binding domain mutation was not significant to ANX2 expression. As a multi-functional protein, HBx was believed to be able to participate a series of signal pathways. As shown in our results, the mutation of SH3 binding domain failed to show significant function in ANX2 level change, this may imply that the HBx does not regulate the ANX2 through a SH3 binding interaction manner.

### 2. Other Differentially Expressed Proteins

In addition to the cytoskeletal and cell migration related proteins, a number of other proteins also showed significant changes. Protein disulfide isomerase A3 (PDIA3) was upregulated in both HepG2 cells transfected with wild type HBV and mutant HBV. The main function of protein disulfide isomerase is to form and break disulfide bonds between cysteine amino acids during protein folding. It is also capable of correcting the wrongly folded proteins to fold in the way of correct arrangement and this function qualifies it as a molecular chaperone. The equal up-regulation in both of the HepG2 cells (with expression of wild type HBV and mutant HBV) may imply that other genes of HBV instead of HBx caused the change. It was reported that protein disulfide isomerase on the cell surface might interact with disulfide bond(s) of glycoprotein 120 (gp120) and such virus-cell fusion facilitated HIV-1 entry [34]. As for HBV, attribute to the absence of an applicable *in vitro* infectivity model, the knowledge of interaction between virus and cell during the early stages of viral infection was very limited. In cancer related research, PDIA3 was found up-regulated in breast cancer tumor tissues compared with normal tissues recently [35].

Another protein that was strongly upregulated was zinc finger CCCH-type antiviral protein 1. It is encoded by gene ZC3HAV1 (also known as ZAP). This protein was known to be functional in preventing retroviruses infection [36]. In the research of rat homolog of this protein, it was found it mainly worked on inhibiting the expression of viral genes. In addition, an innate immune reaction was induced to viral infection. Positional proteomics analysis identified a cleavage of ZC3HAV1 at amino acid residues 260–261 by the HIV-1 protease [37]. It was reported selectively targeting RNA for degradation of HIV [38]. There were also reports showing that zinc finger antiviral protein, together with interferon-induced factor, inhibits alphavirus replication [39]. In HepG2 cells transfected with wildtype HBV genome, this protein showed greatly elevated expression (1.42±0.23). Obviously, the virus expression raised the antiviral activity of the cells. Interferon induced zinc finger antiviral proteins showed high expression level. And in the cells with mutated genome, the level was decreased. However, the mechanism through which that HBx activated interferon via proline rich region still needed to be explored.

In our past work, we had shown the elevated cell deadhesion and cell migration caused by HBV genome expression from the sight of dynamic analysis with the assistance of thermo-responsive polymer material. And it also showed that the mutations of proline rich region effectively restored the elevation. However, the underlying mechanism and signal pathway was not discussed earlier. By applying 2D LC/MS-MS proteomics global data profiling, here we aimed to identify the molecular information that links the proline rich region and cell deadhesion/migration.

**Figure 4:**
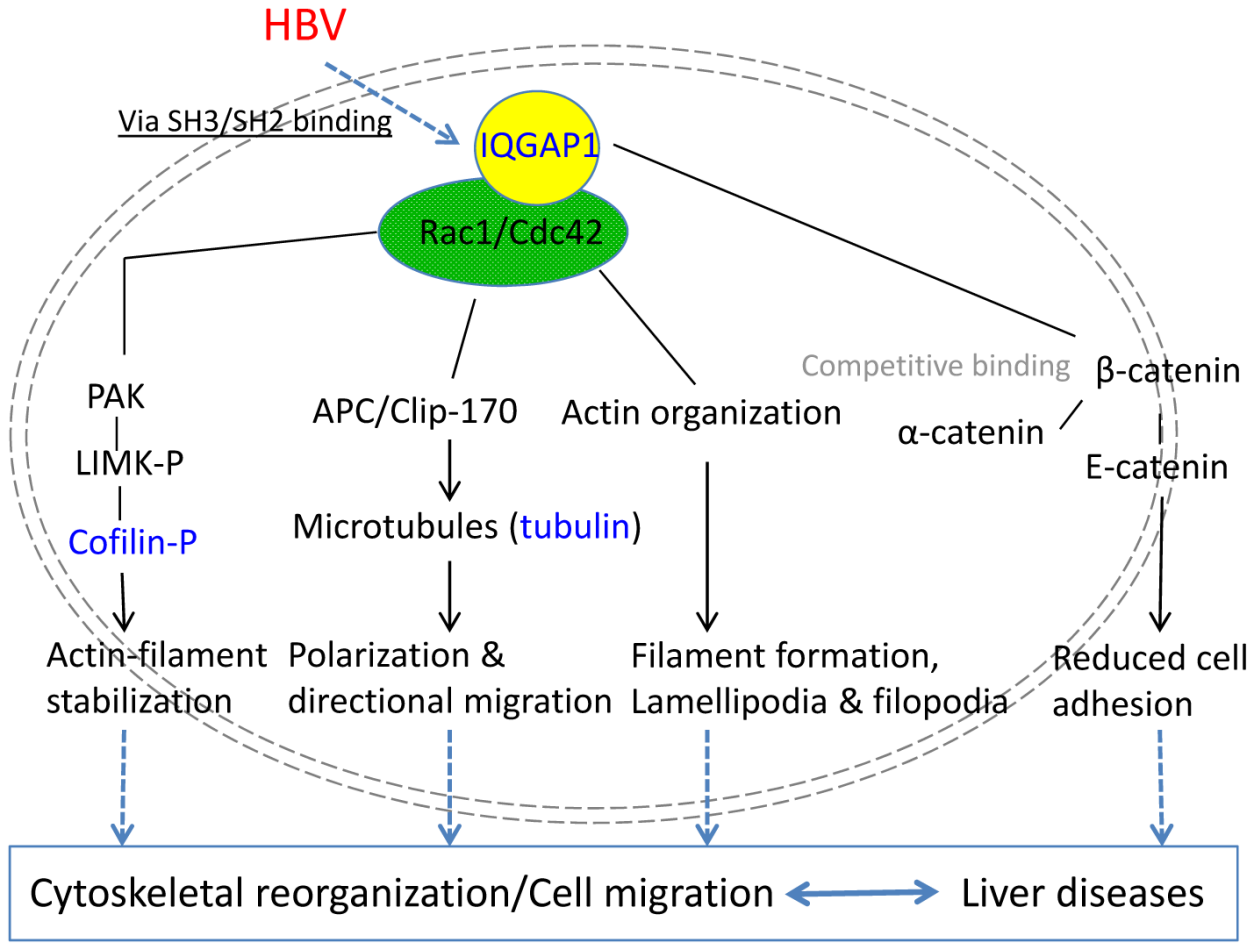
Schematic diagram of HBV and cytoskeletal signaling network. Rho GTPase and downstream signaling effectors protein kinases are involved in cytoskeletal remodeling. Through the interaction with Rac1 and Cdc42, IQGAP1 stabilizes Rho GTPases in GTP bound state. Rac1/cdc42 were believed to influence filament formation, lamellipodia and filopodia by affecting actin organization. Rac1/Cdc42 were also reported to recruit IQGAP1, APC (Adenomatous polyposis coli) and CLIP-170, and the formed complex was a linkage between microtubule and actin cytoskeleton in the progress of cell polarization and directional migration [46]. By the activation Rho GTPases, IQGAP1 indirectly influences actin and tubule cytoskeleton and cell adhesion. IQGAP1 could also directly competitively interact with β-catenin, then E-catenin, which in turn caused decreased cell adhesion.

HBV was believed to target mitochondrial calcium regulation, leading to the activation of Pyk/FAK (Proline-rich tyrosine kinase/Focal adhesion kinase), Src [40], and the signal was then passed into nucleus through Ras-Raf-MAPK-JNK (MAPK, mitogen-activated protein kinase; JNK, Jun N-terminal protein kinase) [41], in turn MMPs (Matrix metalloproteinase) [42]–[44], Myc and Cyclin were trans-activated, resulting the change of cell movements and cell cycle regulation. The change of calcium level could also perturb the level of many calcium binding proteins, such as Annexin and S100 families [45]. HBV was proven to activate Rac1/Cdc42 Rho GTPases, promoting the cell morphology changes [6]. With our LC-MS/MS global protein profiling and gene mutation facilitated results, we found the IQGAP1 showed interesting changes. Considering the function of IQGAP1 played in Rho GTPases activation and cytoskeletal organization, it is possible that HBV signaled through IQGAP1 via specific proline rich region mediated protein-protein interaction to stimulate Rac1 and Cdc42, and the activation of Rac1/Cdc42 could induce a series of cytoskeletal reorganization indirectly, which in consequence reduced cell adhesion and boosted cell migration. On the other hand, IQGAP1 could also directly competitively interact with β-catenin, then E-catenin, which in turn caused decreased cell adhesion (Figure 4).

## Conclusion

In an effort of understanding the mechanism of enhanced cell movements by HBV genome, in addition to our previous observation, we complementarily explored the global protein expression profiling of HepG2 cells expressing HBV genome compared with mutant HBV genome. In our result, a number of proteins including cell migration related proteins were found significantly perturbed by the expression of HBV genome. And such differential expression of those proteins was interestedly found restored to different extent in the HepG2 cells with the expression of proline rich region dis-functioned HBV genome. This may indicate that the differential expression caused by HBV genome was intermediated by the SH3 binding domain located in HBx, considering the fact that the HBV genome with mutated SH3 binding domain was no longer capable of inducing the similar changes. In a certain distance, our proteomics analysis provided more information of induced cell deadhesion, migration and dynamic changes caused by HBV expression presented previously in the perspective of potential intracellular molecular mechanism.
